# BRAF Mutation Analysis in Two Cases of Congenital Self-Healing Langerhans Cell Histiocytosis

**DOI:** 10.7759/cureus.32497

**Published:** 2022-12-14

**Authors:** Saki Takayama, Tadashi Matsubayashi, Masato Koizumi

**Affiliations:** 1 Department of Pediatrics, Seirei Hamamatsu General Hospital, Hamamatsu, JPN

**Keywords:** langerhans cell histiocytosis (lch), oncology pediatrics, spontaneous regression, congenital self-healing langerhans cell histiocytosis, braf mutation

## Abstract

Congenital self-healing Langerhans cell histiocytosis (CSHLCH) is a rare type of Langerhans cell histiocytosis (LCH). Here, we report two cases of CSHLCH. The cases presented solitary and multiple skin lesions of various sizes. The diagnosis was confirmed by skin biopsies. The lesions disappeared after one to two months without therapeutic intervention. No *BRAF* mutations in the skin lesions were detected, and soluble interleukin-2 receptor (sIL-2R) was normal in both cases. Recent studies suggested that the state of differentiation of the precursor cell in which *BRAF* mutations occur is associated with the clinical types and prognosis of the disease. Further investigation should be needed to elucidate the association between the progression and regression of CSHLCH and *BRAF* mutation.

## Introduction

Congenital self-healing Langerhans cell histiocytosis (CSHLCH), also known as Hashimoto-Pritzker disease, is a rare type of Langerhans cell histiocytosis (LCH). The disease has several clinical features, such as incidence in the neonatal period, localization in the skin, and spontaneous regression within a few months. Recent studies on LCH have suggested that the state of differentiation of the precursor cells in which *BRAF* mutations occur correlates with the clinical types and prognosis of the disease [1]. However, there are only a few studies on *BRAF* mutations in patients with CSHLCH. Herein, we reported two cases of CSHLCH and reviewed the literature on *BRAF* mutations in CSHLCH patients.

## Case presentation

First case

**Figure 1:**
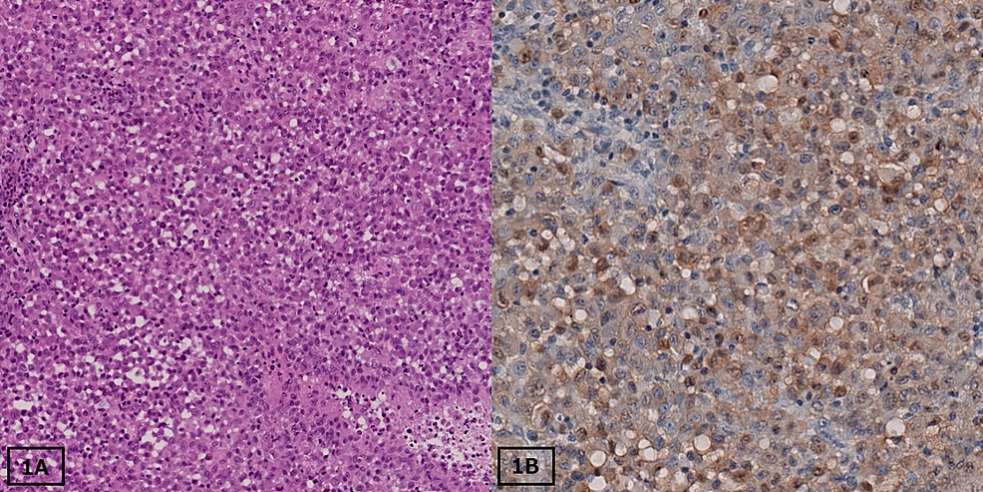
Histopathology of skin biopsy in the first case. (A) On microscopic examination, the tumor revealed extensive infiltration of histiocytic cells with pale eosinophilic cytoplasm and irregular nuclei with hematoxylin and eosin staining at 100× magnification. (B) Tumor cells showed positive staining for S-100 at 200× magnification.

A two-month-old boy was referred to our hospital because of a subcutaneous tumor that grew in the first week of his life. The lesion was protruding, round, and 2.5 cm in diameter with skin ulceration. Physical examination revealed no other abnormal findings, and the patient had no evidence of family history. Additionally, the laboratory values were within the reference range. Soluble interleukin-2 receptor (sIL-2R) was 2,210 U/mL (normal range: 635 to 2,360 U/mL; under two years old). Radiological tests, including full-body X-rays, computed tomography scans, gallium scintigraphy, and bone scintigraphy, were unremarkable. Histological examination of the biopsy specimen revealed extensive infiltration of histiocytic cells with pale eosinophilic cytoplasm and irregular nuclei (Figure 1A). The tumors expressed CD1a, CD68, and S-100 (Figure 1B).

Electron microscopy revealed Birbeck granules. These findings are consistent with those of LCH. The lesion gradually regressed with a scab and disappeared two months later without therapeutic interventions, as previously reported by Hoshino et al. [2]. The patient had no relapse for over seven years.

Second case

**Figure 2:**
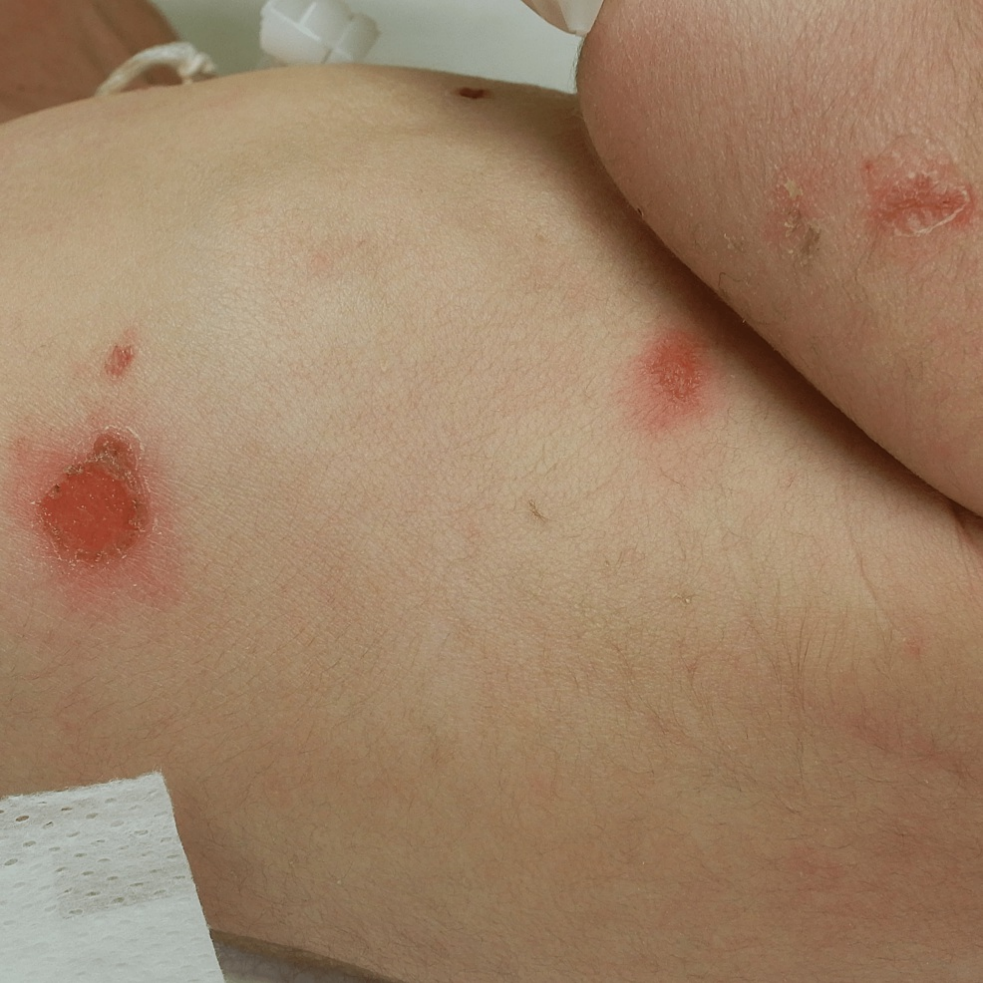
Skin lesions in the second case. Rash and oval erythema with erosion were observed on the left side of the chest, abdomen, and upper extremities.

A zero-day-old boy was transported to the neonatal intensive care unit of our hospital because of a generalized rash at birth. The rash was 2-15 mm in size with oval erythema and erosion (Figure 2).

**Figure 3:**
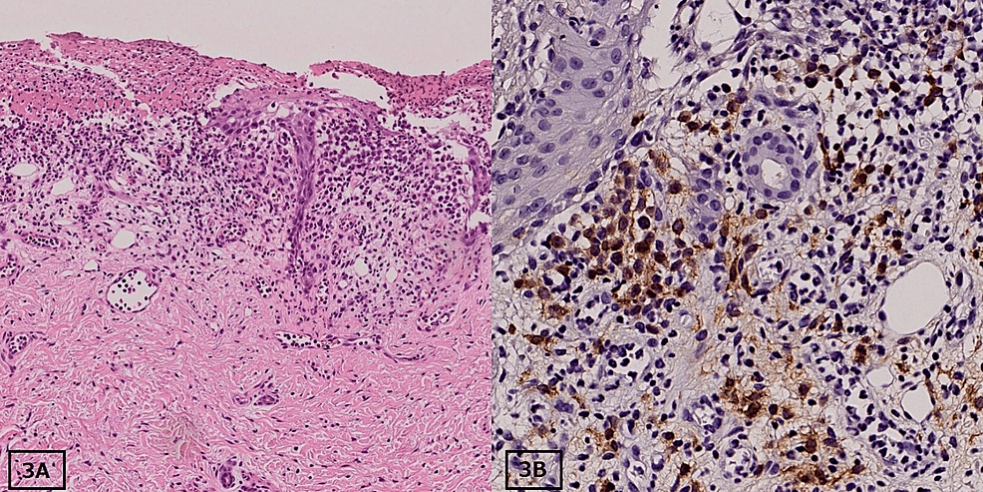
Histopathology of skin biopsy in the second case. (A) Same as the first case, the tumor showed infiltration of ovoid giant cells with atypical nuclei with hematoxylin and eosin staining at 100× magnification. (B) Tumor cells showed positive staining for S-100 at 200× magnification.

The rest of the physical examination was normal. The patient had no relevant family history. The blood laboratory test results were within normal limits. sIL-2R was 1,720 U/mL. Full-body radiography, abdominal ultrasonography, and head magnetic resonance imaging were all normal. A biopsy sample obtained from a skin lesion showed infiltration of ovoid giant cells with atypical nuclei (Figure 3A). These cells were positive for CD1a and S-100 and negative for CD68 (Figure 3B).

These findings led to a diagnosis of LCH. Template DNA was collected from formalin-fixed, paraffin-embedded specimens. *BRAF* V600E was determined using polymerase chain reaction-reverse sequence-specific oligonucleotide as previously described in detail [3]. However, these results were negative. The rash quickly regressed and disappeared a month later without any therapeutic intervention. The patient showed no relapse at one month nor after nine months.

## Discussion

We presented two cases of CSHLCH in which no *BRAF* mutations were found. Their clinical courses were consistent with those of previously reported CSHLCH. LCH can occur at any age, but mostly during childhood. It is characterized by excessive accumulation of clonal mononuclear phagocytes. The sites affected by LCH are highly variable, ranging from a single system, such as the skin or bone, to multi-systemic. In the latter, two or more organs are affected, potentially with risk organ (liver, spleen, and bone marrow) involvement that predicts fatal outcomes. Histology shows granulomatous lesions consisting of pathological Langerhans cells (LCs), lymphocytes, eosinophils, and macrophages. The LCs are characterized by eosinophilic cytoplasm and irregular nuclei with coffee-bean-shaped appearance. Confirmation requires positive staining for CD1a, langerin (CD207), and S-100 with variable expression of CD68 [4].

CSHLCH is a rare type of LCH localized in the skin, presenting at birth or in the neonatal period, although the onset is a few months old in some cases. Cutaneous lesions have asymptomatic red-brown papules or nodules that become ulcerated, necrotic, and then crusted. Approximately 25% of the patients presented with solitary lesions [5]. A noticeable feature is a spontaneous regression within several months. Long-term follow-up is required for patients initially diagnosed with CSHLCH, as previous studies have reported that more than half of the patients had multi-system LCH [6]. Our patients had typical CSHLCH; the onset was within the first month of life, typical histology of LCH, spontaneous regression within a few months, no extracutaneous involvement, and no relapse over several years.

*BRAF* is a member of the mitogen-activated protein kinase (MAPK)/extracellular signal-regulated kinase (ERK) signaling pathway. Recent studies have demonstrated that mutations involving the MAPK/ERK signaling pathway occur in the majority of patients with LCH. The most common mutation in pediatric cases is *BRAF* V600E (50%-60%), followed by MAP2K1 mutation (20%-30%) [7]. However, other rare genetic mutations have also been reported. Identification of the involvement of the activated MAPK/ERK signaling pathway strongly supports that LCH is a neoplastic disorder.

A recent study found that patients with high-risk LCH carried *BRAF* V600E in peripheral CD11c+ dendritic cells, CD14+ monocytes, and bone marrow CD34+ hematopoietic progenitor cells. In contrast, it was found that this mutation was restricted to tissue-resident LCs in low-risk patients, suggesting that *BRAF* V600E acts as a driver mutation [8]. Moreover, Allen et al. described that the state of differentiation of the precursor cell in which *BRAF *mutations occur is associated with clinical type and prognosis [1]. However, there are only a few studies on *BRAF* mutations in patients with CSHLCH. Next, we reviewed the literature on CSHLCH cases tested for *BRAF* mutations (Table 1) [8-16].

**Table 1:** Clinical features of CSHLCH cases analyzed for BRAF mutation. CSHLCH: congenital self-healing Langerhans cell histiocytosis, NR: not recorded.

Author	Number of cases	Age at onset	Number of skin lesion	Time to regression	Positive BRAF mutation
Kansal et al. [8]	1	Birth	Multiple	NR	1
Héritier et al. [9]	6	NR	Solitary	NR	0
Morren et al. [6]	2	Birth	NR	A few wks	0
Schmitt et al. [10]	1	Birth	Multiple	32 d	0
Mori et al. [11]	1	5 wk	Multiple	6 mo	1
Wu et al. [12]	1	5 mo	Multiple	2 mo	1
Nann et al. [7]	3	NR	NR	NR	3
Kaneshima et al. [13]	1	3 mo	Multiple	6 mo	0
Dupeux et al. [14]	5	1 d-2 mo	Solitary	NR	2
Cyr et al. [15]	1	1 d	Multiple	18 mo	0
Rizzoli et al. [16]	1	0 d	Multiple	2 mo	1
This study	2	1 wk, 0 d	Solitary, multiple	3 mo, 2 mo	0

*BRAF *mutations were detected in nine of 25 cases (36%), including our cases. The mutations were found in four out of eight patients (50%) with multiple form lesions and two out of 12 (17%) patients with solitary form lesions. These values were lower than the 77% correlation between multi-system LCH and skin lesions [9]. Kaneshima et al. reported a case of CSHLCH with negative immunostaining for *BRAF* mutation and positive immunostaining for phosphorylated ERK [13], suggesting that activating gene mutations other than *BRAF* may frequently occur in CSHLCH.

However, the mechanism of spontaneous regression in CSHLCH remains unknown. The most plausible mechanism may be the difference in cellular origin between LCH and CSHLCH. Recent studies have demonstrated that LCH lesions arise from self-renewing myeloid dendritic precursors in the bone marrow [1]. Activating gene mutations in the MAPK/ERK signaling pathway causes downregulation of the chemokine receptor CCR7 and upregulation of the anti-apoptotic regulator BCL2L1, resulting in cell senescence and local accumulation rather than proliferation [17]. Moreover, mutated LCs produce numerous cytokines and chemokines that cause inflammation [18]. These processes lead to the development of LCH lesions. Epidermal LC (eLC) precursors migrate from the yolk sac or the fetal liver to the epidermis during the fetal period and undergo sequential differentiation to mature LCs and generate an LC network during the neonatal period [19]. eLCs are highly differentiated and can no longer be supplied from the yolk sac or fetal liver after birth; therefore, they require autonomous replication and proliferation to maintain their population in the epidermis [20]. Similar to myeloid dendritic precursors, mutations in eLCs cause cell senescence, apoptosis suppression, and LCH development. However, mutated eLCs are unable to differentiate, replicate, and proliferate; thus, they eventually die and spontaneously regress. Further investigation is needed to elucidate the association between *BRAF *mutations and CSHLCH progression and regression.

## Conclusions

Herein, we described two cases of congenital self-healing LCH. CSHLCH cases may be underdiagnosed because the lesions are nonspecific and spontaneously regress within several months. Histological examination is required for a definite diagnosis, and analysis of *BRAF* mutations may provide additional information about the clinical type and prognosis of the disease. Additional studies are needed to investigate the correlation between *BRAF* mutations and CSHLCH and to understand the roles of these mutations in disease progression and regression.
